# Bioremediation and Recovery of Lead and Cadmium by Spores of *Bacillus subtilis* C1

**DOI:** 10.1002/mbo3.70170

**Published:** 2025-11-21

**Authors:** Chiara Belaeff, Ylenia De Luca, Luciano Di Iorio, Marina De Stefano, Loredana Baccigalupi, Donato Giovannelli, Ezio Ricca, Anella Saggese

**Affiliations:** ^1^ Department of Biology Federico II University Naples Italy; ^2^ Department of Molecular Medicine and Medical Biotechnology Federico II University Naples Italy; ^3^ Department of Marine and Coastal Science Rutgers University New Brunswick NJ USA; ^4^ Marine Chemistry & Geochemistry Department Woods Hole Oceanographic Institution Woods Hole Massachusetts USA; ^5^ National Research Council—Institute of Marine Biological Resources and Biotechnologies—CNR‐IRBIM Ancona Italy; ^6^ Earth‐Life Science Institute Tokyo Institute of Technology Tokyo Japan

**Keywords:** *Bacillus subtilis*, detoxification, heavy metals, inductively coupled plasma–mass spectrometry

## Abstract

Spores of a hot spring isolated strain of *Bacillus subtilis* were tested as a biotechnological tool to be used for the detoxification and bioremedition of heavy metals. Lead and cadmium were efficiently adsorbed by *B. subtilis* spores with those of C1 more efficient than those of the lab collection strain PY79. Metal‐adsorption did not alter the functionality of C1 spores that were still fully resistant to heat, ethanol or chloroform and able to germinate after the interaction with Cd^2+^ or Pb^2+^. The spore‐adsorbed metals were released upon disruption of the spore coat layers, suggesting that the metals were mostly accumulated within the spore coat. Heat‐inactivated spores released almost all adsorbed metals, allowing the recovery of Cd^2+^ and Pb^2+^. While Cd^2+^ polluted water impaired the normal germination and growth of seeds of the model plant *Arabidopsis thaliana*, treatment of the polluted water with C1 spores restored plant growth.

## Graphical abstract

This study proposes the spore of *B. subtilis* C1 as an efficient detoxification and bioremediation tool. Efficacy of the spore‐based decontamination was tested in vivo with *A. thaliana* seeds. Plant growth was affected by Cd^2+^, but this effect was totally abolished when the contaminated water was treated with C1 spores. The spore‐adsorbed metals can also be efficiently released by either a chemical or physical treatment.

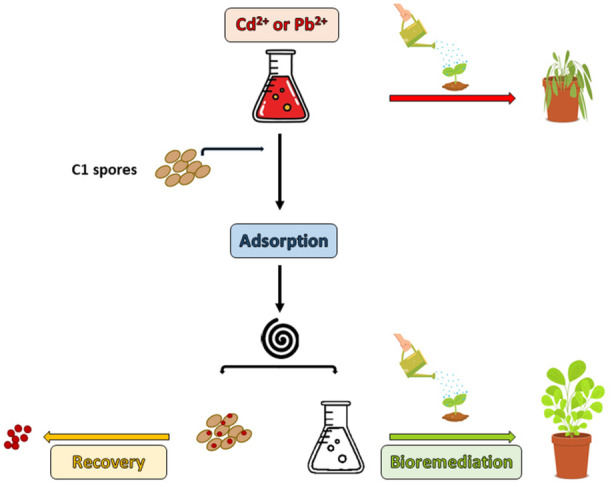

## 1 Introduction

Trace amounts of some heavy metals are essential micronutrients required for biological processes. These include, for example, iron and copper, needed for oxygen and electron transport; cobalt, manganese and nickel, required as enzyme cofactors; or selenium, involved in cell antioxidant activities (Giovannelli 2023; Hay Mele et al. 2023). Other heavy metals, including arsenic, cadmium, mercury and lead, are instead highly toxic, being able to cause various cellular problems, such as the induction of oxidative stress, lipid peroxidation, DNA damage and the inhibition of DNA synthesis (Ooka et al. 2025). Toxic metals accumulate in the environment due to various anthropogenic activities, including industrial processes, vehicle emissions, massive use of fertilisers and incinerator functioning, and thus affect the quality and safety of air, water and soils and cause health issues in plants and animals (Jalilian et al. 2025; Rao and Parsai 2025).

Lead (Pb^2+^) and cadmium (Cd^2+^) salts are two of the most prevalent toxic metal contaminants. Pb^2+^ has been used as a component of gasoline from the 1930s to the early years of the XXI century and, as a consequence, it is now ubiquitously present in the environment at levels estimated to be at least three times higher than those of the preindustrial era (Kumar et al. 2020). In adult humans, the inhalation or ingestion of high levels of Pb^2+^ may cause to cardiovascular, central nervous system, kidney and fertility problems, while they can affect behavior, cognitive performance, postnatal growth, delayed puberty in infants and hamper fetal growth during pregnancy (Kumar et al. 2020). Cd^2+^, when dispersed in the environment as a result of anthropogenic processes, can be carried long distances before it eventually falls in soils or waters. In the soil Cd^2+^ is taken up by plants and is subsequently found in the liver and kidney of animals eating contaminated plants, while in aquatic environments Cd^2+^ is accumulated by filter feeders, such as crustaceans and mollusks, and then passed to fishes feeding on them (Schaefer et al. 2020). As a consequence, all human populations are environmentally exposed to Cd^2+^, mostly through plant‐ or sea‐derived foods. Cd^2+^ has a particularly long half‐life and accumulates in human tissues leading to severe diseases (Schaefer et al. 2020). A positive association between exposure to Cd^2+^ and an increased risk of lung cancer has been established (NTP ‐ National Toxicology Program 2016).

The health‐related risks linked to Pb^2+^ and Cd^2+^ and, in general, to heavy metals, have prompted the scientific community to develop strategies to detect (Sahu et al. 2025; Li et al. 2025) and remove (Malik et al. 2025; Dey et al. 2025) them from the environment. The elimination of heavy metals or the reduction of their concentration from the environment (remediation) can be attempted by many different techniques based on physical or chemical approaches or on the use of living organisms (bioremediation) (Malik et al. 2025). Bioremediation approaches are mainly based on the use of plants (phytoremediation), algae (phycoremediation) or microorganisms (Malik et al. 2025; Makarani and Kaushal 2025; Mallick et al. 2025). In this context, cells of several different microbes have been used to: i) bind metal cations on their surface (biosorption), ii) translocate metal cations in the cytoplasm through metal‐binding proteins, iii) precipitate metal cations by secreted anions or polymers (Xing et al. 2018).

In addition to microbial cells, also bacterial (endo)spores have been used for heavy metals bioremediation (Huang et al. 2013; Lei et al. 2014; Xing et al. 2018). With respect to metabolically active microbial cells, spores are metabolically quiescent and have the advantage of being extremely resistant, even to extreme conditions (McKenney et al. 2013). Such properties are expected to be a relevant factor for both the storage of the prepared bioremediation tool and its field use, where unphysiological conditions, such as nutrient and/or water limitations, high temperatures, low pH and presence of toxic agents, are very likely faced. The spore resistance properties are mostly due to their peculiar structure, characterized by a dehydrated cytoplasm surrounded by a peptidoglycan‐like cortex layer and a proteinaceous coat. Some species also have an additional protective layer made of proteins and glycoproteins (McKenney et al. 2013). Metabolically quiescent spores respond to the presence of water, nutrients and favourable conditions by germinating and originating metabolically active cells able to grow and eventually to sporulate again (Christie and Setlow 2020).

It has been previously reported that spores of a marine *Bacillus* sp. strain (SG‐ 1) efficiently bind manganese (Mn(II)), cobalt (Co(II)) (Lee and Tebo 1994) and copper (Cu(II)) (He and Tebo 1998).

More recently, cells and spores of a *B. coagulans* strain have been shown to efficiently adsorb Pb^2+^ (Xing et al. 2018), while recombinant spores of a laboratory collection strain of *B. subtilis* displaying on their surface a 18‐residue poly‐His tag were shown to bind nickel ions (Hinc et al. 2010).

Here, spores were analyzed for their efficiency in adsorbing Pb^2+^ and Cd^2+^. Spores of C1, a hot‐spring isolated strain of *B. subtilis*, adsorbed both heavy metals more efficiently than spores of a laboratory collection strain of the same species. The spore‐adsorbed metals were released upon heat‐treatment and by extraction of the spore coat proteins allowing the recovery of the adsorbed metals and suggesting their accumulation in the spore coat. Cd^2+^ polluted water once treated with C1 spores, allowed a normal growth of *Arabidopsis thaliana* seeds, thus demonstrating the efficacy of the spore treatment.

## 2 Materials and Methods

### 2.1 Bacterial Strains

PY79 is a well characterized laboratory collection strain of *B. subtilis* (Youngman et al. 1984). C1 is a *B. subtilis* strain isolated from the hydrothermal water of Pisciarelli Solfatara, a hot spring in the center of the Campi Flegrei volcanic complex (40°49045.0768″ N, 14°8 049.3512E). A sample from the hot spring was collected into a sterile bottle and immediately transferred to the laboratory. The sample, mainly consisting of sediments and water, was centrifuged (7000 rpm for 10 min), the pellet was homogenized in sterile Phosphate‐buffered saline (PBS), plated on Difco Sporulation Medium (DSM, for 1 L: 8 g/L Nutrient Broth, 1 g/L KCl, 1 mM MgSO_4_, 1 mM Ca(NO_3_)_2_, 10 μM MnCl_2_, 1 μM FeSO_4_, Sigma‐Aldrich, Darmstadt, Germany) and incubated overnight at 37°C. The recovered colonies were purified by streaking on fresh DSM plates and analyzed for the morphology. Phase‐contrast microscopy was used to analyze spore presence and morphology. C1 strain was selected since its morphological features resembled those of other *B. subtilis* strains. Species assignment was tentatively based on the sequence of the 16S coding gene and then confirmed by genome sequencing.

### 2.2 Spore Purification and Extraction of Spore Coat Proteins

C1 cells were grown in Luria‐Bertani medium (LB; for 1 L: 10 g Bacto‐Tryptone, 5 g Bacto‐yeast extract, 10 g NaCl, pH 7·0) at 37°C for 16 h. Sporulation was induced by exhaustion by growing cells in Difco sporulation medium (DSM) as described previously (Cutting and Vander Horn 1990). After 30 h of incubation at 37°C, spores were collected, washed four times, and incubated overnight in H_2_O at 4°C to lyse residual sporangial cells, as described previously by Nicholson and Setlow (Nicholson and Setlow 1990). The absence of cells after the cold and hypotonic treatment was verified by phase‐contrast microscopy. One aliquot of 1.0 × 10⁸ C1 spores not adsorbed with metals, and other ones adsorbed with cadmium or lead were incubated separately in 50 μl of decoating buffer (0.1 M NaCl, 0.1 M NaOH, 1% SDS) for 30 min at 70°C to extract spore coat proteins (Ragkousi and Setlow 2004). The concentration of extracted proteins was determined by using a Bio‐Rad DC protein assay kit (Bio‐Rad), and 20 ug of total proteins was fractionated on 12.5% SDS‐polyacrylamide gels.

### 2.3 Whole‐Genome Sequencing, Bioinformatic and Pangenome Analysis of C1

Exponentially growing cells were used to extract chromosomal DNA as previously reported (Buglione et al. 2022). Genome sequencing of C1 was performed by GenProbio (Parma, Italy) with Illumina MiSeq Sequencing System and has been deposited in GenBank as BioProject PRJNA1297107 (accession number JBPYUO000000000). Genome assembly was performed with SPAdes v3.9.0 by means of MEGAnnotator pipeline (Lugli et al. 2016). The complete genome sequence of C1 was used for phylogenetic analyses, including several genomes belonging to *B. subtilis* species obtained from the National Center for Biotechnology Information. The phylogenomic trees produced by GTDB‐Tk were visualized on the Interactive Tree of Life (iTOL, platform v6). Average Nucleotide Identity (ANI) values between the sequenced genomes and the closest bacteria were obtained using Ezbiocloud tool (https://www.ezbiocloud.net/tools/ani).

The pangenome of *Bacillus subtilis* C1 was obtained with anvi'o (available from http://github.com/meren/anvio, version 7.1), an open‐source, community‐driven analysis and visualization platform for microbial‐omics. Genes for all genomes were predicted and annotated using Prokka (v 1.14.5) with default parameters. For KEGG Orthology (KO numbers) and COGs database analyses, BlastKOALA was used.

### 2.4 Metal Adsorption Assay

To evaluate the adsorption efficiency of cadmium (^111^Cd) and lead (^208^PB) on the spore surface, a total of 1.0 × 10⁸ spores of *B. subtilis* C1 were incubated with 1, 5, 10 and 100 ppb of these metals at room temperature under continuous shaking. After incubation, the spores were centrifuged for 10 min at 13,000 rpm and the resulting supernatant was filter‐sterilized with a 0.22‐µm filter (Millipore, Bedford, MA, USA). The quantitative analysis of metals in the supernatant, corresponding to the non‐adsorbed fraction, was performed by ICP‐MS (Agilent 7900). Before analysis, 1% nitric acid was added to each sample to reach a final volume of 15 mL, which was required for triplicate measurements by the instrument, according to Correggia et al. 2024. Metal concentrations provided by the instrument are expressed in ppb (1 ppb corresponding to 1 µg/L). Each experiment was conducted in duplicate.

### 2.5 Physiological Analysis of C1 Spores

#### 2.5.1 Heat Resistance

Suspensions of spores adsorbed with heavy metal and spores alone (1.0 × 10⁸) were subjected to heat treatment at 80°C for 15 min. Following this incubation, the samples were serially diluted and plated onto 2% LB agar plates. The plates were then incubated overnight at 37°C to determine colony‐forming units (CFUs).

#### 2.5.2 Organic Solvent Resistance

Suspensions of 1.0 × 10⁸ spores without metals or adsorbed with cadmium or lead were incubated at room temperature with ethanol and chloroform 10% for 10 min and then the suspensions were serially diluted and plated on LB agar (2%) plates and incubated overnight at 37°C for CFU count.

#### 2.5.3 Germination Efficiency

Purified spores (1.0 × 10⁸) were heat activated (30 min at 70°C), and germination was induced by adding the indicated germinants. Germination was measured by OD_580_ decrease by using as the germinant 10 mM l‐asparagine or 10 mM l‐alanine in a buffer containing 10 mM Tris‐HCl (pH 8.0) and a buffer containing 1 mM fructose, 1 mM glucose, and 10 mM KCl. The optical density at 580 nm was measured at 5‐min intervals for 30 min. (Saggese et al. 2016). Each experiment was conducted in triplicate.

### 2.6 In vivo effects

To assess the effectiveness of the spore‐based treatment in the decontamination of Cd²⁺‐polluted water, we evaluated the effects of cadmium‐contaminated water on the growth of *Arabidopsis thaliana* seedlings. Particular attention was given to root development, as it is known to be more sensitive to the toxic effects of heavy metals (Lee et al. 2021). The *Arabidopsis thaliana* Columbia‐0 (Col‐0) ecotype was used as a model plant in this study. Seeds were surface sterilized using 70% ethanol for 2 min, followed by rinsing in sterile distilled water to remove any residual ethanol.

40 seeds of wild plants were placed on sterile Murashige and Skoog (MS) solid soil as a control, another 40 seeds were placed on MS soil added with water contaminated with 5000 ppb of Cadmium, and finally, another 40 seeds were placed on MS soil added with the supernatant resulting from the treatment of cadmium with C1 spores. The seeds were germinated in a growth chamber with conditions of 16 h light/8 h dark at 20°C‐22°C. After 4 days, root growth was assessed by measuring it with a ruler.

### 2.7 Statistical Analysis

Data were reported as mean values ± SEM. The Graph PadPrism 10 program (GraphPad Software, San Diego, CA, USA) was used to perform one‐way ANOVA followed by the Dunnett's post‐hoc test. A probability of < 5% (*p* < 0.05) was considered statistically significant in all analysis.

## 3 Results

### 3.1 Adsorption of Pb^2+^ and Cd^2+^ By *Bacillus subtilis* Spores

**Figure 1:**
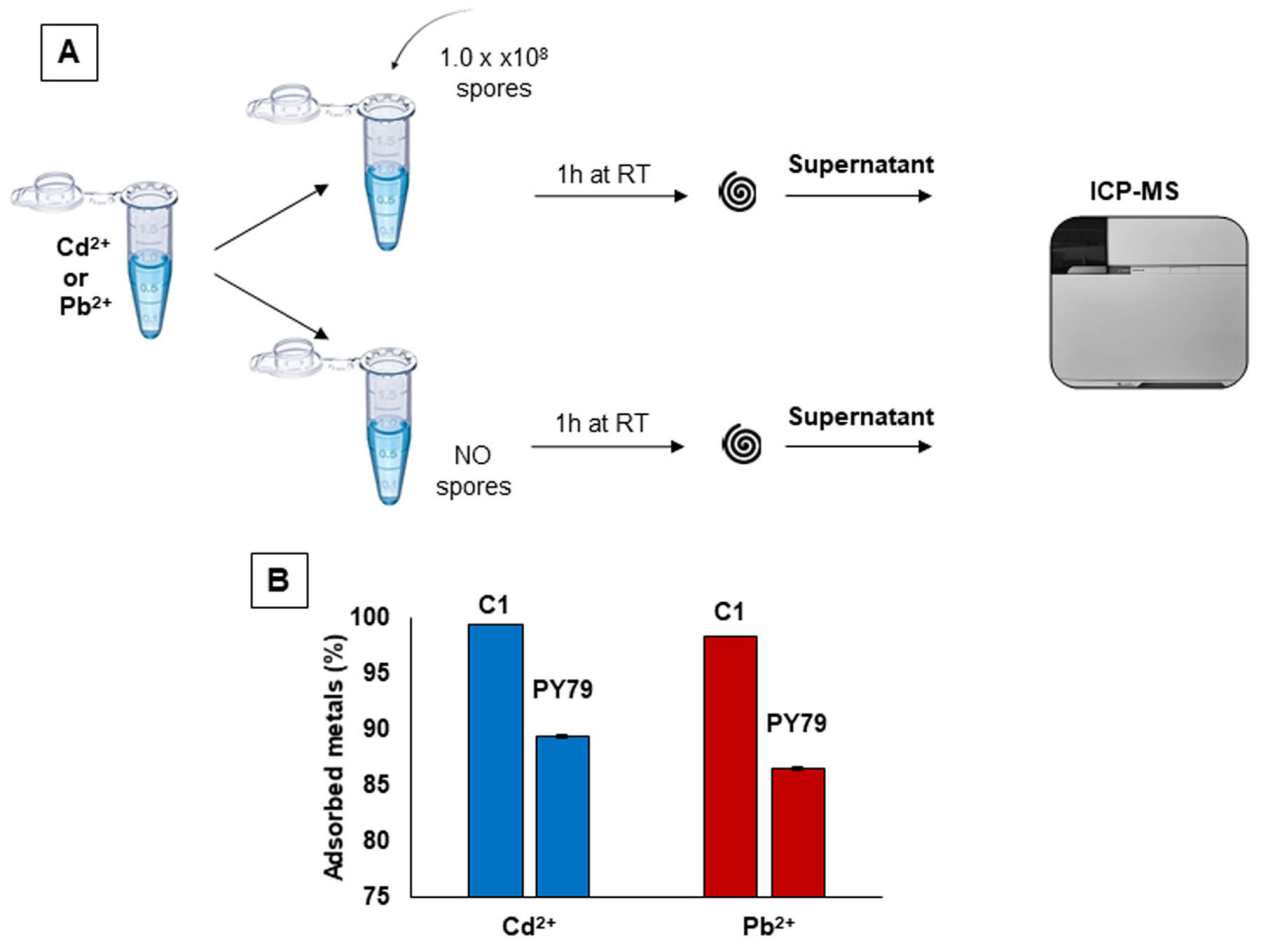
(A) Schematic representation of metal adsorption. (B) Metal adsorption by *B. subtilis* C1 and *B. subtilis* PY79 spores. The amount of adsorbed metals is given as the percentage of the spore bound ppb (initial ppb minus the ppb left in solution). Data represent mean ± standard deviation of two biological samples each measured in triplicate by ICP‐MS.

The ability of spores to adsorb Cd^2+^ and Pb^2+^ was evaluated by Inductively Coupled Plasma—Mass Spectrometry (ICP‐MS) as schematically reported in Figure 1A. Water solutions of 5 ppb (parts per bilion) (corresponding to 5 μg/L) of Cd^2+^ and 10 ppb (corresponding to 10 μg/L) of Pb^2+^ were prepared. These concentrations were selected as they are indicated as the maximum permissible levels of Cd^2+^ and Pb^2+^ for drinking water by the European law (Directive (EU) 2020/2184). Each solution was splitted in two halves and 1.0 ×10^8^ spores added to one half for each metal. Tubes were incubated at room temperature (RT) for 1 h, fractionated by centrifugation and the supernatant fractions filter‐sterilized and analyzed by ICP‐MS (Figure 1A). The second half of each metal was treated identically but without the addition of spores (Figure 1A).

Spores of two strains were used: C1, a hot‐spring isolated strain tentatively assigned to the *B. subtilis* species on the base of the sequence of its 16S RNA coding gene (not shown), and PY79, a well‐characterized laboratory collection strain (Youngman et al. 1984).

The amount of metals measured in the supernatant fractions are reported in Table 1. Spores of both strains adsorbed most of the Cd^2+^ and Pb^2+^ present in solution and only minimal amounts of heavy metals were left in the supernatant fractions after the incubation with spores. However, the amount of metal found in the supernatant fractions was higher after the incubation with PY79 than with C1 spores (PY79/C1 ratio in Table 1), indicating the latter spores as more efficient in metal adsorption. The percentages of metals adsorption of spores of the two strains are reported in Figure 1B. For their higher efficiency of adsorption, C1 spores were selected and used for all further analysis.

**Table 1:** Cd^2+^ and Pb^2+^ (ppb) in the supernatant fractions without and with C1 and PY79 spores.

Heavy metal a		NO spores^b^	C1 spores^b^	PY79 spores^b^	PY79/C1 ratio
Cd^2+^	5.0	4.806 ± 0.067	0.030 ± 0.010	0.512 ± 0.094	17.06
Pb^2+^	10.0	9.823 ± 0.399	0.082 ± 0.061	1.355 ± 0.098	16.52

^a^ Amount weighted (μg/L).

^b^ Measured by ICP‐MS. Each experimental value is the average of two biological replicates each independently measured three times.

### 3.2 Genomic Analysis of *B. subtilis* C1

The whole genome sequence of the C1 strain was obtained with a coverage of ~30×, with 72 contigs (Suppl. Mat. Table S1). A total of 4.189 CDS, 16 rRNA and 75 tRNA‐coding genes were assigned (Suppl. Mat. Table S1). The obtained genome is approximately 4.0 Mbp long with a 43.7% GC content (Suppl. Mat. Table S1). No plasmid sequences were detected (not shown). The Average Nucleotide Identity (ANI) of the genome was determined against the genomes of two type strains of *B. subtilis* and showed values of 98.75% and 98.77%, versus strain 168 and NCIB 3610, respectively, confirming the previous tentative assignment based only on the 16S sequence.

**Figure 2:**
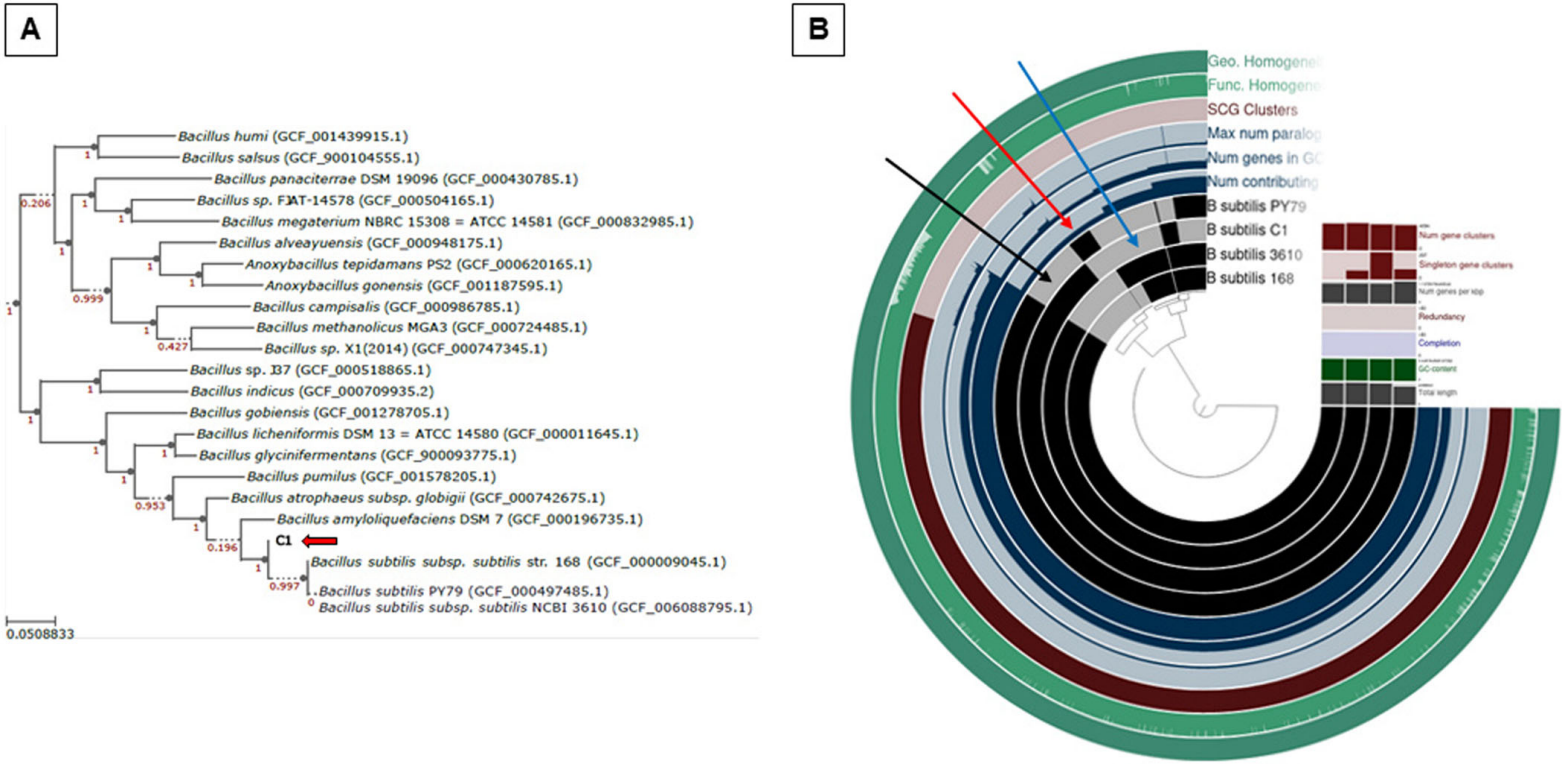
(A) Phylogenetic tree based on the C1 genome sequence (red arrow) constructed using the GTDB‐Tk tool. The tree was visualized on the Interactive Tree of Life (iTOL, platform v6). (B) Anvi'o representation of the pangenome of the 4 *B. subtilis* genomes generated with the items order in presence/absence (D: Euclidean; L: Ward). Each black layer represents the genome of a strain. The additional layers indicate the single‐copy gene (SCG) clusters (present once in each genome) and the GC content. The unique region present on C1 chromosome is indicated by the black arrow; the regions absent in C1 genome, but present in PY79 or in other strains are indicated by red and blue arrows, respectively.

The phylogeny of the C1 strain was further analyzed by comparing its genome with those of several other strains of the *Bacillus* genus. A phylogenetic tree, obtained as described in Material and Methods, clearly confirmed that C1 belongs to the *B. subtilis* species (Figure 2A).

A pangenomic analysis (Figure 2B) showed that C1 shares large parts of its genome with those of PY79, 168 and NCIB 3610 strains. However, an over 300 Kbps region is present on the C1 chromosome but not on that of the other strains considered in this analysis (black arrow in Figure 2B) while other regions present in PY79 (red arrow in Figure 2B) or in other strains (blue arrow in Figure 2B) are lacking in C1. The over 300 Kbps of DNA present only on the C1 chromosome code for about 300 putative products listed in Suppl. Mat. Table S2.

### 3.3 C1 Spores Adsorb High Concentrations of Pb^2+^ and Cd^2+^ (Figure 3)

**Figure 3:**
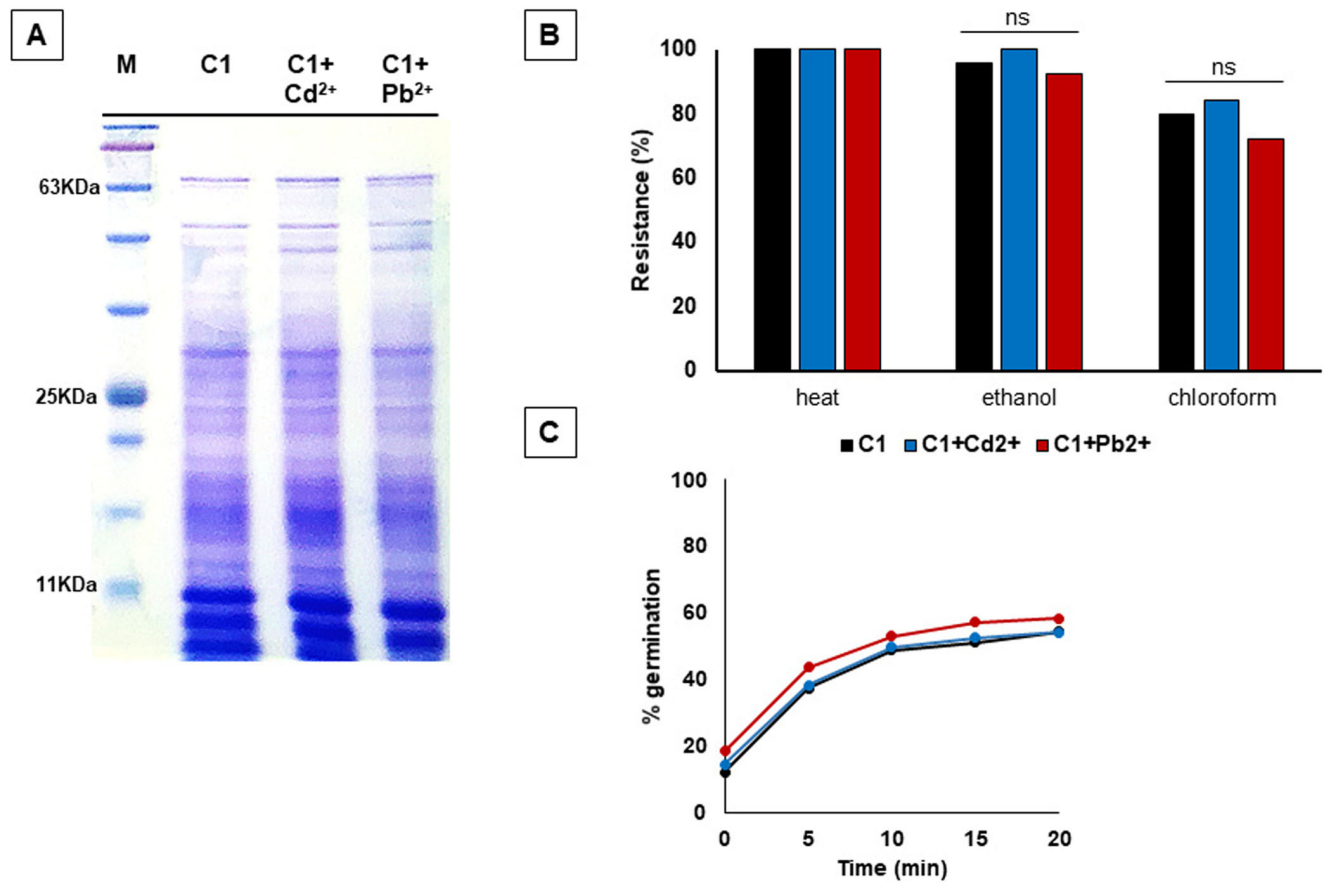
(A) SDS‐PAGE of protein samples extracted from C1 spores with NaOH/SDS treatment. Samples (20 µg per lane) were separated on a 12.5% acrylamide gel and stained with Comassie Brilliant Blue. Lane 1: molecular weight marker (kDa indicated on the left); Lane 2: C1 spores not adsorbed with metals; Lanes 3 and 4: spores adsorbed with 5 ppb of Cd2+ or 10 ppb of Pb2+ respectively. (B) Resistance of spores adsorbing heavy metals to heat, ethanol and chloroform. C1 spores without metals (black bars) or adsorbed with cadmium (blue bars) or lead (red bars) were compared. (C) Germination efficiency of C1 spores (black curve) and spores with adsorbed cadmium (blue curve) and lead (red curve). The germination was induced using l‐Ala‐GFK and measured as the percentage of loss of the OD_600_.

To better define the metal adsorption efficiency of C1 spores various concentrations of both Cd^2+^ and Pb^2+^ were tested. In addition to the concentrations indicated as the maximum permissible levels of Cd^2+^ and Pb^2+^ for drinking water tested in Table 1 (5 and 10 ppb for Cd^2+^ and Pb^2+^, respectively), lower and higher concentrations for each metal were used. As reported in Table 2, at all concentrations of Cd^2+^ or PB^2+^ tested (between 1 and 100 ppb) only minimal amounts of metals were found not adsorbed in the supernatant fractions with an efficiency of adsorption always over 97%.

**Table 2:** Cd^2+^ and Pb^2+^ (ppb) in the supernatant fractions without and with C1 spores.

Heavy metal^a^		NO spores^b^	C1 spores^b^	Not adsorbed ppb (%)	Adsorbed ppb (%)
Cd^2+^	1.0	0.914 ± 0.046	0.012 ± 0.008	1.4	98.6
	5.0	4.806 ± 0.067	0.030 ± 0.010	0.6	99.4
	10.0	9.368 ± 0.186	0.167 ± 0.016	1.8	98.2
	100.0	107.053 ± 2.620	1.156 ± 0.078	1.1	98.9
Pb^2+^	1.0	1.109 ± 0.005	0.033 ± 0.032	3.0	97.0
	5.0	4.979 ± 0.021	0.102 ± 0.015	2.1	97.9
	10.0	10.185 ± 0.050	0.166 ± 0.008	1.6	98.4
	100.0	103.235 ± 0.309	1.307 ± 0.244	1.3	98.7

^a^ Amount weighted (μg/L).

^b^ Measured by ICP‐MS. Each experimental value is the average of two biological replicates each independently measured three times.

The experiments summarized in Table 2, then indicate that C1 spores adsorb over 97% of water‐suspended Cd^2+^ or Pb^2+^ when their concentration ranges between 1 and 100 ppb. Based on this, all further experiments were performed with a Cd^2+^ concentration of 5 ppb and a Pb^2+^ concentration of 10 ppb corresponding to the maximum permissible levels of Cd^2+^ and Pb^2+^ for drinking water by the European law (Directive (EU) 2020/2184).

### 3.4 C1 Spores Adsorbed with Pb^2+^ and Cd^2+^ Are Fully Functional

Adsorption of Cd^2+^ or Pb^2+^ did not affect the viability of C1 spores and the same number of colony forming units (CFUs) were observed when 1.0×10^8^ free or metal‐adsorbed spores (estimated by microscopy count with a Burker chamber) were plated on LB medium (not shown). To assess whether the adsorption of the toxic heavy metals affected the structure or the functionality of C1 spores, purified spores adsorbed with 5 ppb of Cd^2+^ or 10 ppb of Pb^2+^ were compared with free (not adsorbed with metals) spores for their profile of extracted spore surface proteins and their resistance‐germination properties. C1 spores were purified as described previously (Nicholson and Setlow 1990) and coat proteins extracted by treatment with NaOH/SDS (Ghosh et al. 2008). Extracted proteins were fractionated by SDS‐PAGE and no differences were observed when Cd^2+^ or Pb^2+^ were adsorbed before Cot protein extraction (Figure 3A), suggesting that the presence of the adsorbed metals did not influence the spore coat structure or the extraction of Cot proteins.

Purified spores of C1 were then tested for their resistance to a heat‐treatment and to the presence of ethanol or chloroform. In all cases, free‐ and metal‐adsorbed spores were equally resistant indicating that the presence of adsorbed heavy‐metals did not alter spore resistance and recovery of viable bacteria (Figure 3B).

Spore germination was evaluated by monitoring the OD_580nm_ variations of a spore suspension upon heat‐activation and supplementation of l‐alanine (l‐Ala) or l‐asparagine (l‐Asn) as germinants, as previously reported (Saggese et al. 2022). C1 spores efficiently germinated in response to l‐Ala and the adsorption of either Cd^2+^ or Pb^2+^ did not affect the germination response (Figure 3C). Surprisingly, C1 spores did not respond to l‐Asn as germinant, independently from the presence of Cd^2+^ or Pb^2+^ (not shown). Analysis of the C1 genome indicated the presence of the *gerA* and *gerK* operons, while the *gerB* locus lacked of the third gene *gerBC* (Suppl. Mat. Table S3). Since *gerA* products are required for the response to l‐Ala while *gerB* and *gerK* products cooperate to allow germination in response to l‐Asn (Zhang et al. 2025), failure to respond to l‐Asn by C1 spores is likely due to the lack of *gerBC* gene.

### 3.5 Pb^2+^ and Cd^2+^ Mostly Accumulate in the C1 Spore Coat

**Figure 4:**
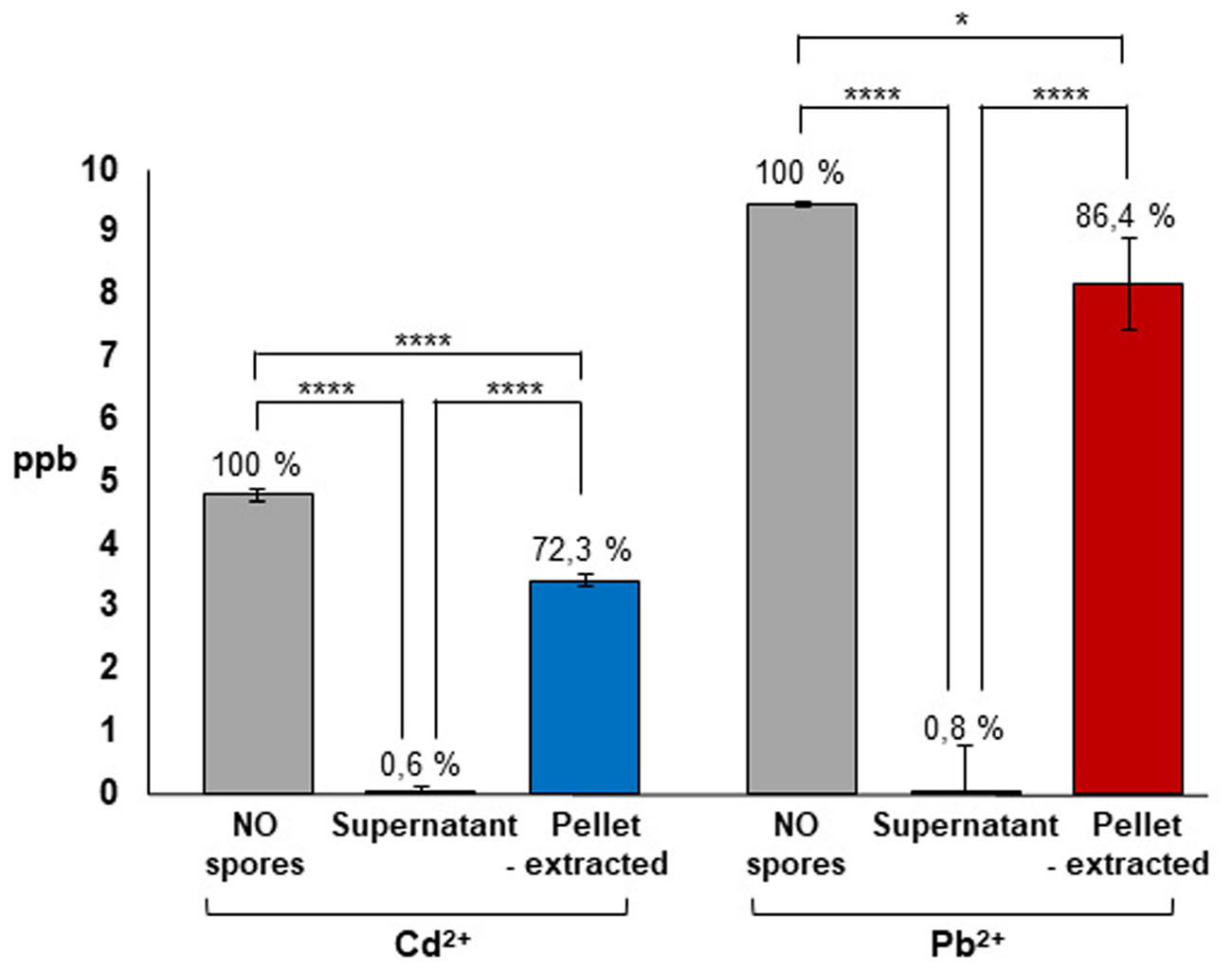
Release of spore‐adsorbed metals by chemical treatment. For each metal the initial ppb (No spores), the unbound ppb (Supernatant) and the released ppb (Pellet‐extracted) are reported. Data represent mean ± standard deviation of two biological samples each measured in triplicate by ICP‐MS. Statistical differences among groups were evaluated by one‐way ANOVA.

Upon adsorption with spores Cd^2+^ and Pb^2+^ could either be attached to the spore surface or translocate across the surface and localize in the inner parts of the spore. To address this point, spores were adsorbed with either 5 ppb of Cd^2+^ or with 10 ppb of Pb^2+^ as indicated in Figure 1A. The pellet fractions were used to extract the spore surface proteins (Cot proteins) as for the experiment of Figure 3A and analyzed by ICP‐MS for the presence of metals in parallel with the supernatant fractions and with the metal solutions without spores. In agreement with the results reported in Table 2, only minimal amounts of metals were found in the supernatant fractions (Figure 4). The pellet fractions contained over 70 and over 80% of Cd^2+^ and Pb^2+^, respectively (Figure 4), indicating that the large majority of the adsorbed metals was on the spore surface, associated with the Cot proteins.

### 3.6 The Adsorbed Metals are Released by Heat Treatment

**Figure 5:**
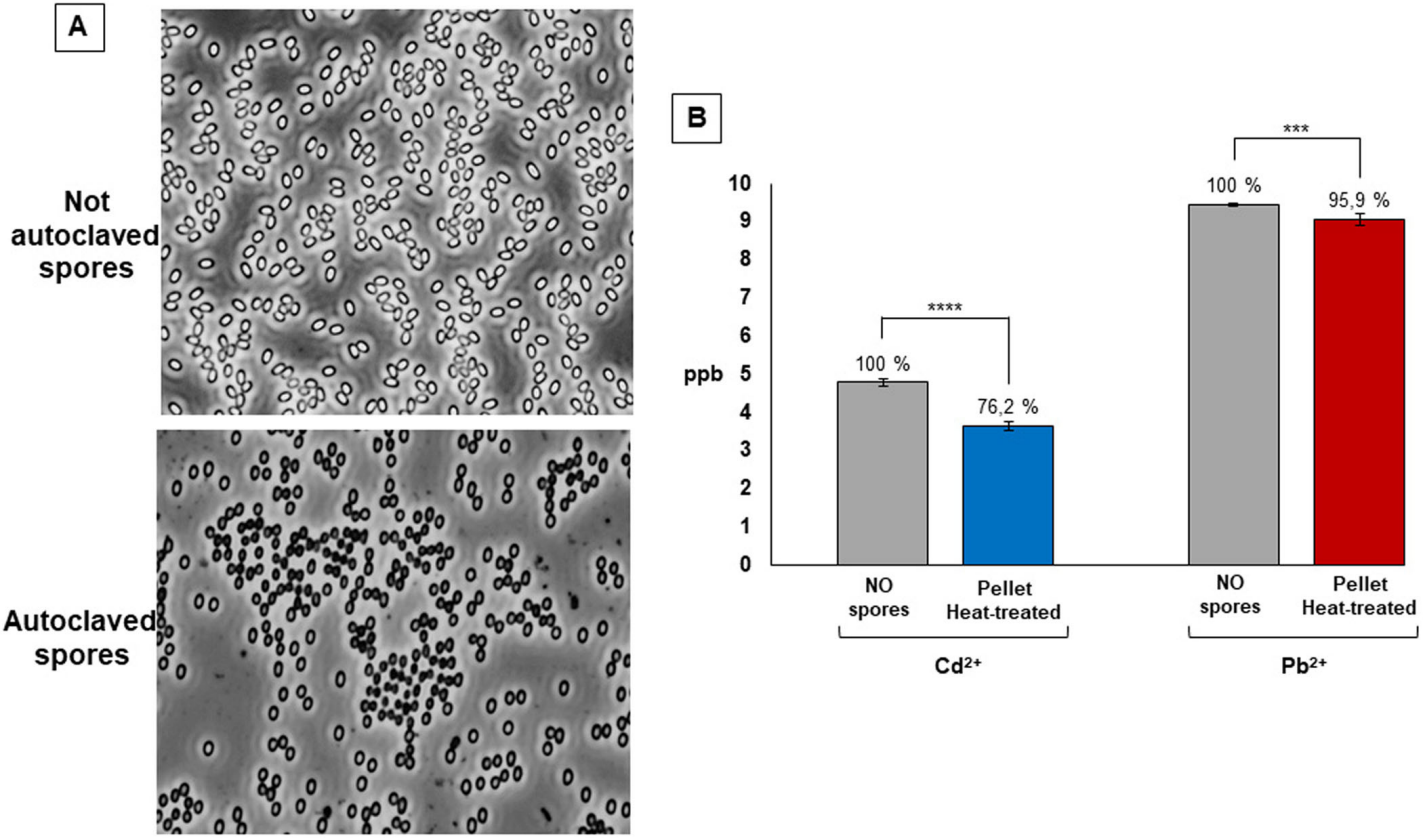
(A) C1 spores observed under the phase‐contrast microscope before (top panel) and after treatment at 121°C for 10 min in autoclave (bottom panel). (B) Release of spore‐adsorbed metals by heat‐treatment. For each metal the initial ppb (No spores) and the released ppb (Pellet Heat‐extracted) are reported. Data represent mean ± standard deviation of two biological samples each measured in triplicate by ICP‐MS. Statistical differences among groups were evaluated by T‐test.

Results of Figure 4 also indicated that the adsorbed metals could be released, allowing the recovery of the metals (bioremediation). Since NaOH and SDS were used to extract the Cot proteins, those chemicals were also present in the metal solution. To avoid the presence of such chemicals the release of the adsorbed metals was attempted by heat treatment. Spores were adsorbed with either 5 ppb of Cd^2+^ or with 10 ppb of Pb^2+^ as indicated in Figure 1A and the pellet fractions re‐suspended in water and heat‐treated in autoclave at 121°C for 10 min. The heat‐treatment caused a strong decrease of spore viability (from 1.0 × 10^8^ to 2.6 × 10^2^ CFU before and after the treatment) and most spores appeared damaged under the light microscope (Figure 5A). However, cell debris were not evident by light microscopy after the heat‐treatment (Figure 5A) and the heat‐treated spores once fractionated by centrifugation did not released proteins (< 0.03 μg/μl protein in solution by Bradford assay; Bradford 1976). The heat‐treated spores were fractionated by centrifugation and the supernatant filtered and analyzed by ICP‐MS for the presence of metals in parallel with the metal solutions without spores. The supernatant fractions contained over 76 and over 90% of Cd^2+^ and Pb^2+^, respectively (Figure 5B), indicating that the heat‐treatment was slightly more effective than the coat‐protein extraction for the release of the adsorbed metals.

### 3.7 The Spore‐Treatment Reduces the Toxic Effects of Cd^2+^ in Vivo

**Figure 6:**
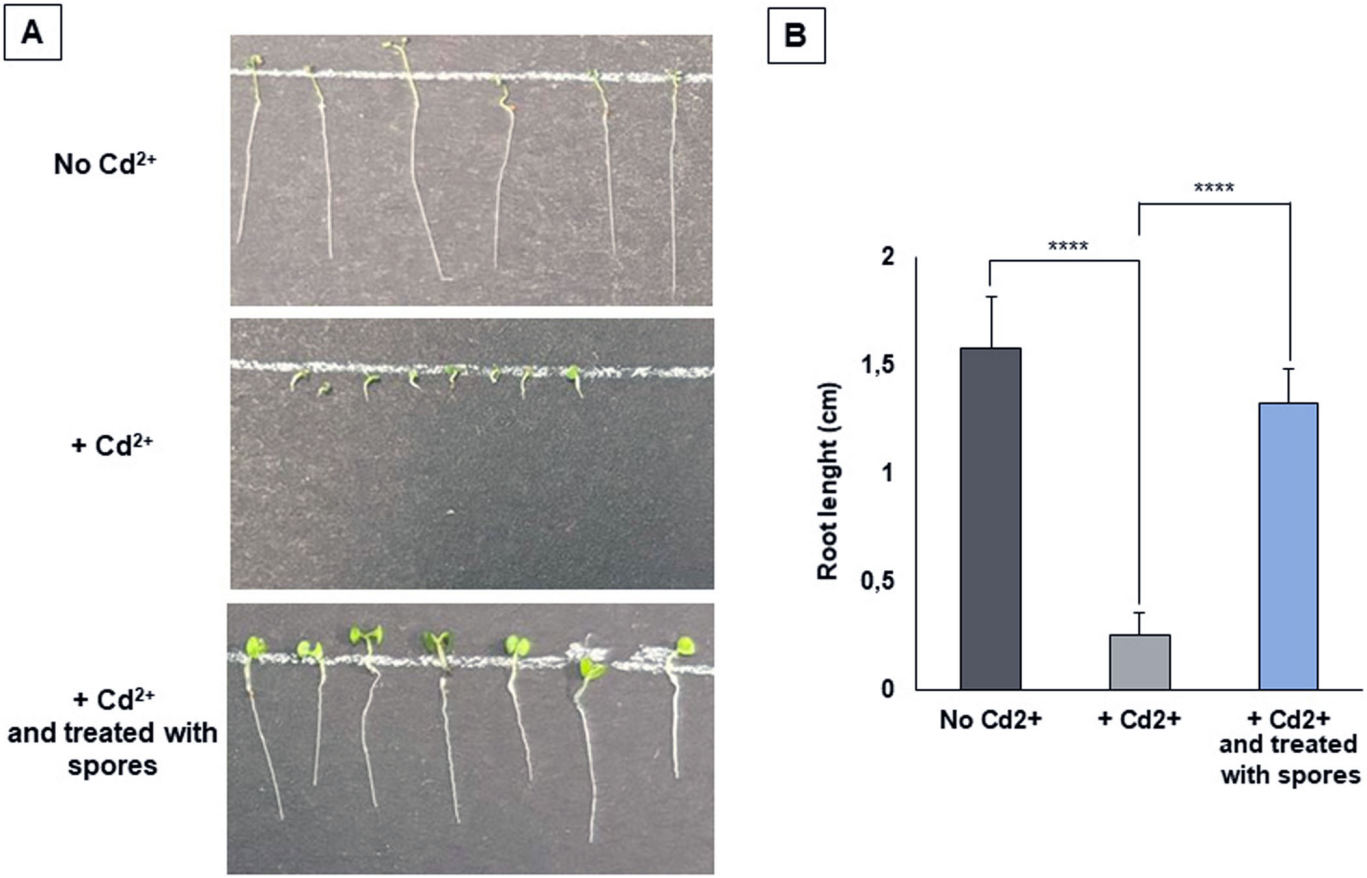
Growth of *Arabidopsis thaliana* seeds with no cadmium, with cadmium or with cadmium‐polluted water treated with C1 spores. (A) Representative images of some *A. thaliana* seeds. All images were taken 4 days after seed germination. (B) Lenght of primary root (cm) after 4 days of growth of 40 seeds for each condition. Statistical differences were assessed using a two‐tailed t‐test (*p* < 0.05).

To assess the efficacy of the spore‐treatment in the decontamination of polluted water, the effects of Cd^2+^ polluted water were analyzed on the growth of the model plant *Arabidopsis thaliana*. To set up the experimental conditions, several concentrations of Cd^2+^ were tested and a water solution containing 5000 ppb of the metal was needed to strongly inhibit plant growth (Figure 6A). This concentration is 1000‐fold higher than that used in most of the previous experiments of this study. As reported in Table 2, 1.0 × 10^8^ spores were able to adsorb 99% of the Cd^2+^ present in a water solution containing 100 ppb of the metal. For the adsorption of 5000 ppb of Cd^2+^ a 100‐fold higher number of spores (1.0 × 10^10^) was used. The polluted water once treated with C1 spores allowed a normal growth of *A. thaliana* seeds (Figure 6A) as indicated by a statistical analysis of 40 seeds for each condition (Figure 6B).

## 4 Discussion

This study proposes the spore of *B. subtilis* C1 as an efficient detoxification and bioremediation tool. Spores are metabolically quiescent and resistant to conditions that would be lethal for other cells (Park and Ramamurthi 2025; Riley et al. 2020), therefore C1 spores can be considered for field use where lack of nutrients and harsh conditions are likely encountered. C1 spores efficiently adsorb Cd^2+^ and Pb^2+^ and accumulate most of the adsorbed metals in the spore coat. This is not surprising since the spore coat is mostly composed of proteins (McKenney et al. 2013) and metals efficiently bind thiolic, amino and carboxylic groups of amino acids (Tamás et al. 2014). In many cases metals‐protein interactions cause protein misfolding and degradation (Tamás et al. 2014), but this doesn't seem to occur with the interaction of Cd^2+^ and Pb^2+^ with the *B. subtilis* Cot proteins. Although the Cot protein profile of free and metal‐adsorbed spores was analyzed by SDS‐PAGE and SDS denature proteins, in many cases folded and unfolded proteins behave differently on SDS‐PAGE due to aggregation and high rates of proteolysis of misfolded proteins (Tiwari et al. 2019). The apparently unaltered profile of the Cot proteins on SDS‐PAGE, together with the lack of any effect of the spore viability, resistance or germination indicate that the adsorption of Cd^2+^ and Pb^2+^ does not damage C1 spores.

The spore‐adsorbed metals can be efficiently released by either a chemical (Cot protein extraction) or physical (heat) treatment, with the latter being slightly more efficient. While the chemical treatment caused the release of soluble Cot proteins and metals, the heat‐treatment cause the release of the adsorbed metals but not the solubilization of the Cot proteins. A possible explanation of these results is that most metals bind functional groups of the amino acid residues of the Cot proteins and only part of them (less than 30%) are either bound to insoluble (not extracted) Cot proteins or reach the inner parts of the spore. When soluble Cot proteins are released, most metals (over 70%) are also released. Upon heat treatment, Cot proteins are not released but are probably denatured and unable to keep the metals bound. Further experiments will be needed to clarify this point.

Efficacy of the spore‐based decontamination was tested in vivo with *A. thaliana* seeds (Auzane et al. 2025). Plant growth was not affected by various concentration of Pb^2+^ while it was affected by 5,000 ppb of Cd^2+^. The Cd‐dependent effect on *A. thaliana* growth was totally abolished when the contaminated water was treated with C1 spores, confirming in vivo the potentials of C1 as a detoxification system.

## 5 Conclusions

C1 is a hot‐spring isolated spore former assigned by genomic analysis to the *Bacillus subtilis* species. C1 spores adsorb Cd^2+^ and Pb^2+^ more efficiently than a laboratory collection strain of the same species and the interaction with either metal does not affect spore survival or functionality. Most of the adsorbed metal localize in the spore coat and can be released by a chemical or physical treatment. Results reported in this study point to C1 spores as a powerful tool for the detoxification of polluted environments and the recovery (bioremediation) of the contaminating metals.

## Author Contributions

**Chiara Belaeff:** investigation (lead), validation (lead), visualization (lead). **Ylenia De Luca:** investigation (supporting), validation (supporting), visualization (supporting). Marina De Stefano: investigation (supporting). **Luciano Di Iorio:** investigation (supporting), validation (supporting), sotware. **Donato Giovannelli:** writing review and editing (supporting), resources (supporting). **Loredana Baccigalupi:** writing review and editing (supporting), **Ezio Ricca:** writing original draft, resource (lead). **Anella Saggese:** supervision, writing review and editing (lead). All authors read and approved the final manuscript.

## Conflicts of Interest

The authors declare no conflicts of interests.

## Ethics Statement

The authors have nothing to report.

## Supporting information


**Table S1:** General features of C1 genome. **Table S2:** Genes and putative products present only in strain C1. **Table S3:** Genes coding for receptors involved in germination found in C1 and percentage of identity with proteins encoded by the reference strain B. subtilis 168.

## Data Availability

The data that support the findings of this study are available in the supporting information of this article.
